# Supplementary material for: Modeling glioblastoma heterogeneity as a dynamic network of cell states
Source: Mol Syst Biol. 2021 Sep 16;17(9):e10105. doi: 10.15252/msb.202010105 (PMC8444284; doi:10.15252/msb.202010105)
Supplement: Supplementary file 6 — Source Data for Figure 5 [file MSB-17-e10105-s004.zip › Figure5A_sourcedata/GSEA_3017/nosigdb_stateA.GseaPreranked.1622795026485/DOROSZKO_U3054MG_CLUSTER_2.html]

Details for gene set DOROSZKO\_U3054MG\_CLUSTER\_2[GSEA]

|  || Dataset | state53017 |
| Phenotype | NoPhenotypeAvailable |
| Upregulated in class | na\_neg |
| GeneSet | DOROSZKO\_U3054MG\_CLUSTER\_2 |
| Enrichment Score (ES) | -0.47493926 |
| Normalized Enrichment Score (NES) | -2.562113 |
| Nominal p-value | 0.0 |
| FDR q-value | 5.882353E-5 |
| FWER p-Value | 0.001 |
Table: GSEA Results Summary

  

Fig 1: Enrichment plot: DOROSZKO\_U3054MG\_CLUSTER\_2      
 Profile of the Running ES Score & Positions of GeneSet Members on the Rank Ordered List

  

| PROBE | GENE SYMBOL | GENE\_TITLE | RANK IN GENE LIST | RANK METRIC SCORE | RUNNING ES | CORE ENRICHMENT || 1 | PDGFRA |  |  | 5 | 0.871 | 0.0246 | No |
| 2 | IGFBP3 |  |  | 26 | 0.641 | 0.0252 | No |
| 3 | TGIF1 |  |  | 107 | 0.447 | -0.0453 | No |
| 4 | ZEB1 |  |  | 109 | 0.446 | -0.0310 | No |
| 5 | LPAR4 |  |  | 184 | 0.387 | -0.0971 | No |
| 6 | EPB41L2 |  |  | 208 | 0.371 | -0.1090 | No |
| 7 | NR2F2 |  |  | 303 | 0.324 | -0.1987 | No |
| 8 | FARP1 |  |  | 332 | 0.315 | -0.2180 | No |
| 9 | GAD1 |  |  | 423 | 0.288 | -0.3046 | No |
| 10 | C11orf96 |  |  | 494 | 0.271 | -0.3704 | No |
| 11 | EBF1 |  |  | 519 | 0.267 | -0.3870 | No |
| 12 | VEGFA |  |  | 546 | 0.261 | -0.4059 | No |
| 13 | FZD2 |  |  | 551 | 0.259 | -0.4013 | No |
| 14 | SOX9 |  |  | 577 | 0.254 | -0.4194 | No |
| 15 | DNM1 |  |  | 592 | 0.251 | -0.4258 | No |
| 16 | NES |  |  | 603 | -0.252 | -0.4278 | No |
| 17 | NR3C1 |  |  | 610 | -0.256 | -0.4255 | No |
| 18 | BST2 |  |  | 614 | -0.259 | -0.4198 | No |
| 19 | PLIN3 |  |  | 619 | -0.259 | -0.4152 | No |
| 20 | RGS16 |  |  | 620 | -0.260 | -0.4062 | No |
| 21 | SHROOM3 |  |  | 639 | -0.264 | -0.4165 | No |
| 22 | SIPA1L1 |  |  | 645 | -0.267 | -0.4127 | No |
| 23 | SEZ6L2 |  |  | 672 | -0.283 | -0.4308 | No |
| 24 | EFEMP2 |  |  | 686 | -0.294 | -0.4346 | No |
| 25 | STIM2 |  |  | 694 | -0.298 | -0.4319 | No |
| 26 | TMEM59L |  |  | 698 | -0.299 | -0.4248 | No |
| 27 | ZFHX4 |  |  | 700 | -0.301 | -0.4155 | No |
| 28 | MDFIC |  |  | 709 | -0.308 | -0.4135 | No |
| 29 | EMP1 |  |  | 724 | -0.322 | -0.4175 | No |
| 30 | KCNE4 |  |  | 758 | -0.348 | -0.4410 | No |
| 31 | TSPAN13 |  |  | 760 | -0.349 | -0.4300 | No |
| 32 | BOC |  |  | 766 | -0.359 | -0.4230 | No |
| 33 | IGFBP7 |  |  | 780 | -0.368 | -0.4243 | No |
| 34 | SRGAP1 |  |  | 799 | -0.383 | -0.4305 | No |
| 35 | MRC2 |  |  | 813 | -0.395 | -0.4308 | No |
| 36 | BNIP3L |  |  | 841 | -0.432 | -0.4450 | No |
| 37 | SDCBP |  |  | 845 | -0.438 | -0.4331 | No |
| 38 | DAAM1 |  |  | 885 | -0.516 | -0.4572 | Yes |
| 39 | MEG3 |  |  | 894 | -0.537 | -0.4473 | Yes |
| 40 | ARHGAP29 |  |  | 899 | -0.548 | -0.4328 | Yes |
| 41 | PLK2 |  |  | 901 | -0.551 | -0.4149 | Yes |
| 42 | ITGB5 |  |  | 903 | -0.558 | -0.3968 | Yes |
| 43 | DKK3 |  |  | 912 | -0.579 | -0.3854 | Yes |
| 44 | CYTOR |  |  | 918 | -0.591 | -0.3705 | Yes |
| 45 | FRMD4B |  |  | 925 | -0.626 | -0.3554 | Yes |
| 46 | ID1 |  |  | 931 | -0.651 | -0.3384 | Yes |
| 47 | CHN1 |  |  | 934 | -0.680 | -0.3171 | Yes |
| 48 | CLU |  |  | 938 | -0.685 | -0.2968 | Yes |
| 49 | LRRC17 |  |  | 949 | -0.769 | -0.2811 | Yes |
| 50 | COL5A2 |  |  | 958 | -0.856 | -0.2602 | Yes |
| 51 | CDK6 |  |  | 966 | -0.920 | -0.2361 | Yes |
| 52 | CDKN1A |  |  | 969 | -0.987 | -0.2043 | Yes |
| 53 | CTGF |  |  | 976 | -1.182 | -0.1701 | Yes |
| 54 | ID3 |  |  | 981 | -1.505 | -0.1226 | Yes |
| 55 | SPARCL1 |  |  | 984 | -1.810 | -0.0625 | Yes |
| 56 | CCL2 |  |  | 985 | -1.881 | 0.0021 | Yes |
Table: GSEA details [plain text format]

  

Fig 2: DOROSZKO\_U3054MG\_CLUSTER\_2: Random ES distribution      
 Gene set null distribution of ES for **DOROSZKO\_U3054MG\_CLUSTER\_2**
